# Study on the Effect of Blood Content on Diffuse Reflectance Spectra of Basal Cell Carcinoma Skin Tissue

**DOI:** 10.1155/2013/192495

**Published:** 2013-08-19

**Authors:** Miaoqing Nan, Qingli He

**Affiliations:** Department of Physics, Northwest University, Xi'an, Shaanxi 710069, China

## Abstract

Diffuse reflectance spectrum as a noninvasive method has been widely used to study the optical properties of cutaneous skin tissue. In this work, we optimized an eight-layered optical model of basal cell carcinoma skin tissue to study its optical properties. Based on the model, the diffuse reflectance spectra were reconstructed in visible wavelength range by Monte Carlo methods. After different blood contents were added to the optical model, the contribution of blood to diffuese reflectance spectra was investigated theoretically. The ratios of basal cell carcinoma skin and normal skin tissue were also calculated for both experimental result and rebuilt results to testify the theoretical reasonability of the model and methods.

## 1. Introduction

Diffuse reflectance spectrum carries the information from the organization and has been investigated to study the optical properties of skin tissue, and analysis of malignant biological tissue components of skin diseases and pathogenesis [1, 2]. The diffuse reflectance at each wavelength will escape to the skin surface which was observed. Biological properties of skin tissue could be analyzed and classified by studying *in vivo* diffuse reflectance spectra experimentally.

Basal cell carcinoma (BCC) is a type of nonmelanoma skin cancer, which most commonly occurs in Caucasians. Reports show that long-term sun exposure, the environment of high ambient solar irradiance, ultraviolet (UV) ray, skin color, and race are related to BCC cancer [3]. BCC is rarely fatal, which is most commonly located on the head and neck, but the incidence is increasing rapidly with age [4]. In recent years, a large number of research works focused on the BCC clinical treatment [4, 5]. As BCC has an intrinsically low optical contrast, there is not a noninvasive method for the detection and delineation direct of this tumor as we know [6].

The Monte Carlo (MC) simulation has been developed to solve the problem of light transportation in biological tissues [7]. MC method and its specific simulation processes have been introduced in detail [8]. A simplified seven-layer skin optical model for normal skin has been reported previously [1, 9, 10]. The model of non-melanoma skin cancer was rare. In this study, we built an eight-layer optical model to study the BCC skin reflectance spectra in the visible wavelength range. In order to find the contribution of blood content to the reflectance spectra, different blood factors were added into the optical model. Based on MC simulation calculation, we modeled the diffuse reflectance process from BCC skin tissue. We also measured the reflectance spectra of normal skin and BCC skin *in vivo* to be compared with the simulated spectra. It is believed that this modeling work will be helpful for a further study on BCC skin optical properties.

## 2. Methods

### 2.1. Experimental System Setup

The setup of *in vivo* diffuse reflectance spectra measurement has been described in details in [11]. A quartz tungsten halogen lamp was employed as light source. A fiber bundle consists of seven fibers with one center for light collection and the other surrounding six for illumination. A specially designed skin probe is used to prevent the specular reflection and the pressure effect. 

**Figure 1:**
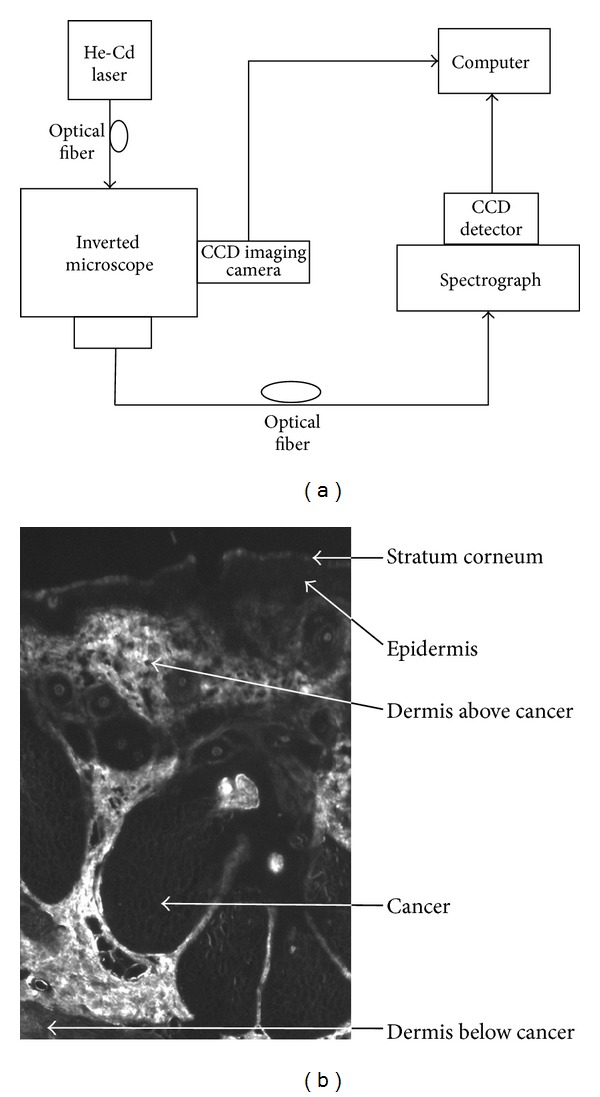
(a) The system setup of MSP system. (b) The fluorescence spectra of different skin layers measured from a 16 *μ*m unstained BCC tissue section at 442 nm excitation.

In our previous study, we have obtained a typical autofluorescence image of a BCC skin tissue section by a microspectrophotometer (MSP). The MSP system and autofluorescence image of different skin layers were show in Figure 1. The MSP has been described in details in [12]. Fresh cutaneous tissue samples were frozen and cut parallel to the tissue surface of 16 *μ*m thickness. A 442 nm He-Cd laser output was used to illuminate and excite the unstained samples. From the image, BCC regions and their surrounding areas have a sharp boundary. BCC regions were dark and nonfluorescent, surrounded by brighter regions. We also found that there were strip-like structures within the BCC skin. The dermis layer exhibited higher autofluorescence intensity than the layer of stratum corneum and epidermis. Based on the autofluorescence image, we attempt to build an optical model of BCC skin. The BCC layer is located below the upper blood plexus but on the top of the dermis layers.

### 2.2. Optical Model

As human skin has a complex structure, a simplified seven-layer model of normal skin was developed for theoretical simulation [7, 9].  Table 1 outlines this model and provides the thickness (*d*), refractive index (*n*), and the optical transport parameters (absorption coefficient, *μ*_*a*_, scattering coefficient, *μ*_*s*_, and scattering anisotropy, *g*) at 442 nm for each skin layer. The transport parameters of the stratum corneum, epidermis, and dermis were compiled by Graaf et al. [13].

The upper blood plexus was modeled as a uniform layer consists of 10% blood and 90% dermis tissue. The lower blood plexus which consists of 90% blood and 10% dermis tissue. The optical parameters (*μ*_*a*_, *μ*_*s*_, *g*, *n*) of these two layers were calculated by adding the fractional contributions of the blood and dermis components [14]. The tissue optical parameters were calculated by the following matrix formula [7, 9]:

(1) $$\begin{array}{c} \left[\begin{array}{c} \mu_{a}\\ \mu_{s}\\ g\\ n \end{array}\right]=[\begin{array}{c} \mu_{a}^{1}\\ \mu_{s}^{1}\\ g^{1}\\ n^{1} \end{array}\begin{array}{c} \mu_{a}^{2}\\ \mu_{s}^{2}\\ g^{2}\\ n^{2} \end{array}]\cdot \left[\begin{array}{c} f_{1}\\ f_{2} \end{array}\right], \end{array}$$

where *f*_1_ and*f*_2_ are the percentages of each component, respectively. Superscripts 1 and 2 in the first matrix on the right side, respectively, indicate the optical properties of components 1 and 2. For example, the refractive index of the upper blood plexus was estimated to be *n* = 1.4 × 0.90 + 1.33 × 0.10 = 1.39.

According to the fluorescence spectra of different skin layers in Figure 1, we intend to build an optical model of BCC skin. We modified the BCC skin optical model to an eight-layer model by adding a new layer into the model, show in Table 2. The BCC layer is located between the upper reticular dermis and reticular dermis, with a thickness of 400 **μ*m*. The thickness of the reticular dermis reduces from 1500 **μ*m* to 1100 **μ*m*, and the total thickness of the skin model is still 2 mm. The absorption coefficient and scattering coefficient of BCC tissue were compiled by Elena Salomatina et al. [6]. By using integrating sphere spectrophotometry combined with the inverse MC technique, they measured some skin samples from the face, neck, and back of the patients. The refractive index and anisotropy factor of the BCC layer were 1.4 and 0.8 [6]. Thus, we established an eight-layer BCC skin optical model in Table 2. In order to find the contribution of blood content to the reflectance spectrum, we added different blood content (2%, 5%) into the BCC layer. The optical parameters were calculated by formula (1). Tables 3 and 4 list the corresponding optical parameters for 2% and 5% blood content added to the optical model. In order to simulate the fluorescence escape process, we also require the transport parameters at an other visible wavelength range. 

### 2.3. The Monte Carlo Simulation Method

In our simulation, the Monte Carlo code [8, 15] was used to calculate the diffuse reflectance to reconstruct the diffuse reflectance spectra of normal skin, BCC skin tissue and BCC skin tissue with different blood factor (2% and 5%) added to the BCC layer directly. From 480 to 700 nm in 10 nm intervals, a total of 23 data points were generated to form each reflectance spectrum curve. In each simulation, 1,000,000 photons were launched. We also use Monte Carlo code to calculate the excitation light distribution inside the model skin.

## 3. Results and Discussion

**Figure 2:**
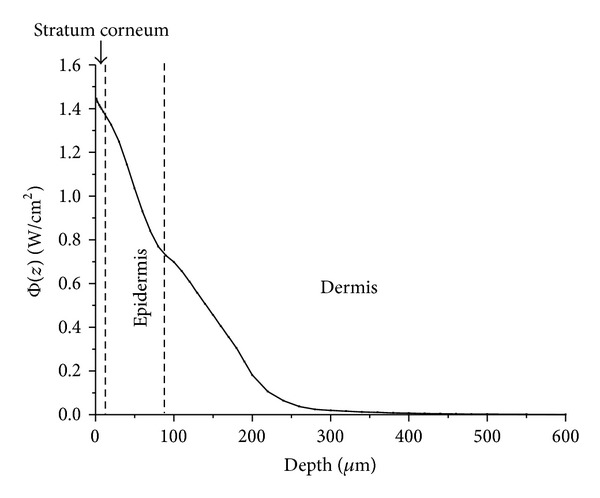
The excitation light (442 nm) distribution as a function of depth *z* inside the BCC skin tissue (5% blood factor added to the BCC layer).

We used the Monte Carlo simulation to calculate the 442 nm exciting light transmission and distribution in BCC skin tissue without blood factor being added inside.  Figure 2 shows the excitation light distribution inside the model skin as a function of depth *z*, in this model, 5% blood was added to the BCC layer as an example. It can be seen that light intensity decreases fast with increased depth *z*, only a small amount of light transport into the lower dermis. Excitation light was mainly distributed in the stratum corneum, epidermis layer and the papillary dermis, they contribute the most to diffuse reflectance spectra.

**Figure 3:**
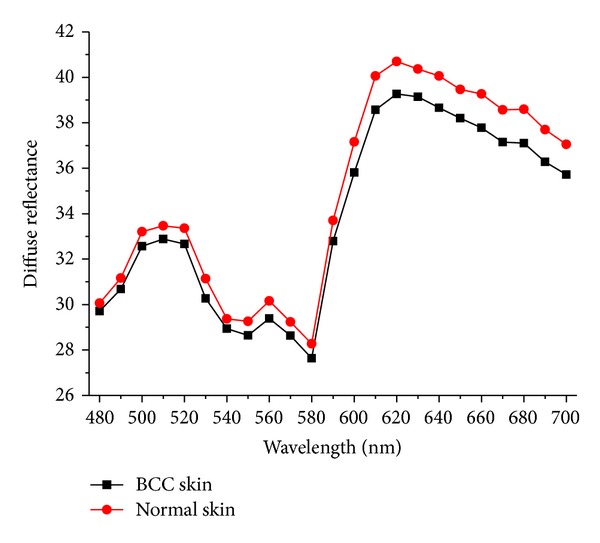
The measured reflectance spectra of BCC skin and normal skin.

Figure 3 shows the measured reflectance spectrum of BCC skin and normal skin by *in vivo* diffuse reflectance spectral measurement system. The reflectance spectra showed similar shape but varied intensity between BCC skin and normal skin. Normal skin has higher reflectance intensity than BCC skin. Below 600 nm, the BCC curve fits quite close to the normal skin curves. While being 600 nm to 700 nm wavelength, the spectra intensity was different. According to the effect of absorption by blood, the diffuse reflectance spectral of both normal skin and BCC skin tissue appeared double absorption valleys of blood at 540 nm and 580 nm.

**Figure 4:**
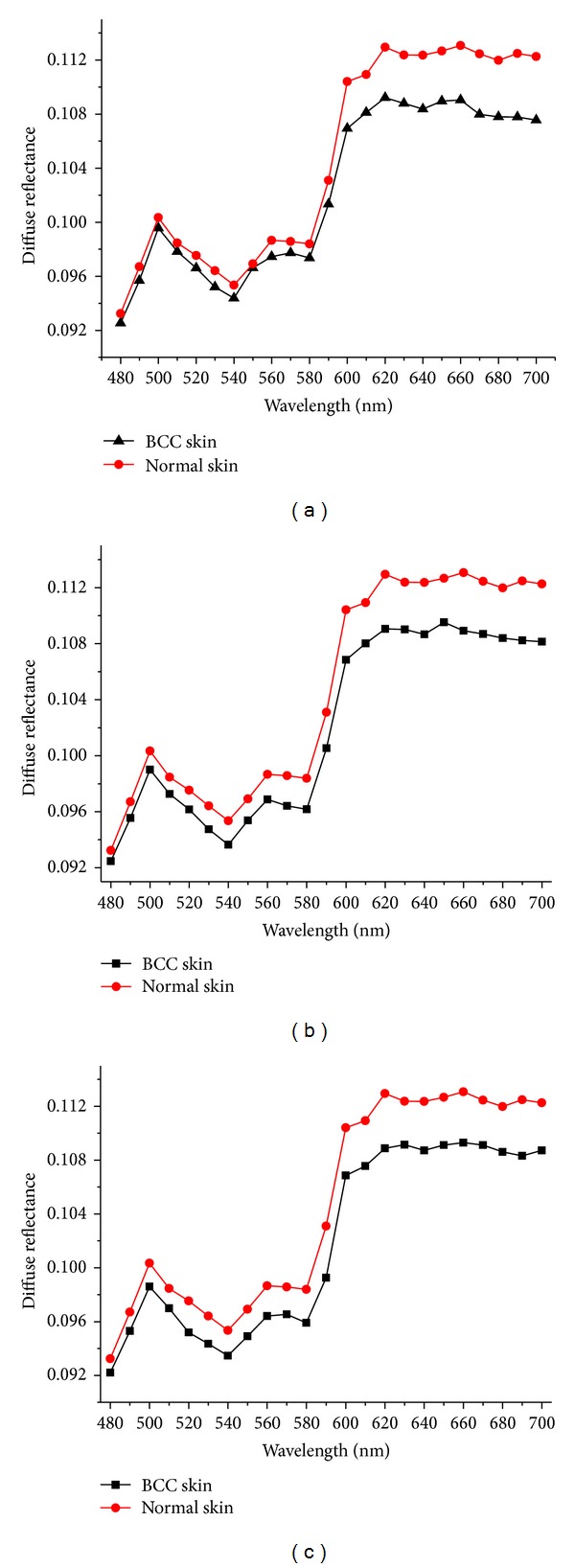
(a) Monte Carlo calculated reflectance spectra of BCC skin. (b) Monte Carlo calculated reflectance spectra of BCC skin. (2% blood factors added to the BCC layer). (c) Monte Carlo calculated reflectance spectra of BCC skin. (5% blood factors added to the BCC layer).

Figure 4 shows the calculated reflectance spectra of BCC skin with different blood factors being added inorder to be compared with calculated reflectance spectrum of normal skin. It can be seen that, reflectance spectra calculated by MC simulation have a good agreement with the experimental results. The calculated reflectance spectra also showed similar shape but varied intensity between BCC skin and normal skin. With contrast these three results in Figure 4, the blood content of BCC layer has an effect on the intensity of reflectance spectra. Below 600 nm, the spectra intensity of BCC curve decrease as the absorption and scattering of tissues increases with more blood being added. Although different blood contents are added to optical models, normal skin has higher reflectance intensity than BCC skin. This is mainly due to the fact that the BCC layer that we added to skin model has a faster decreased absorption coefficient in long wavelength range, but for normal skin, the absorption coefficient of dermis layer changes little in longer wavelength range. 

**Figure 5:**
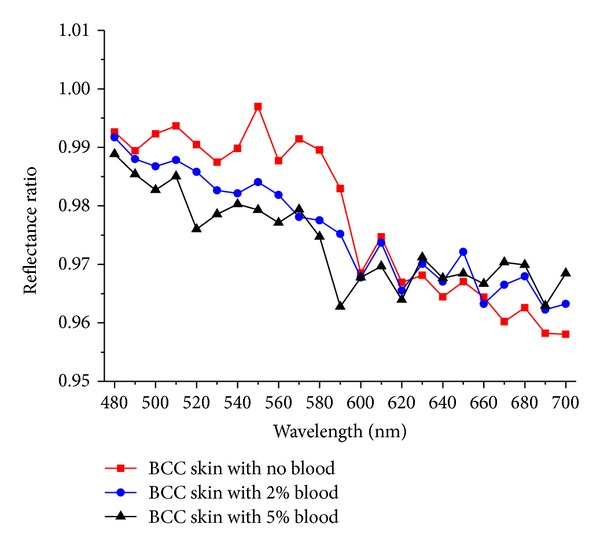
Calculated spectral ratio curves of rebuilt results.

Figure 5 shows the calculated spectral ratio curves of rebuilt results of different blood contents added to the optical model. Reflectance ratio curve consists of BCC skin spectral intensity divided by the normal skin spectral intensity. The spectral shape of the reflectance ratio curves by MC simulation was determined by blood content in BCC layer. The reflectance ratio curve of BCC skin with no blood added was higher than others. 

**Figure 6:**
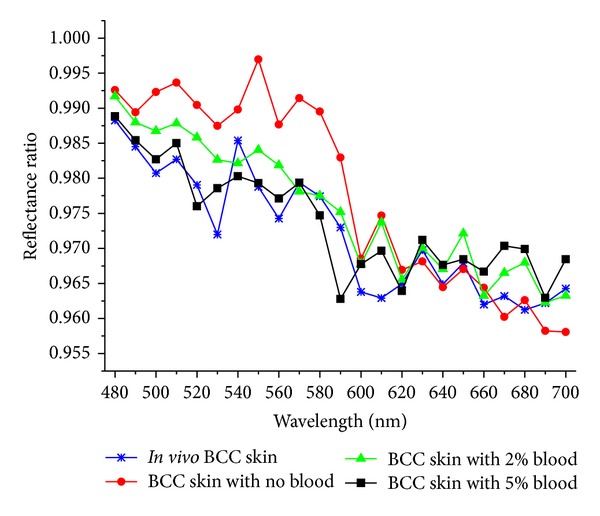
Calculated spectral ratio curves of experimental result and rebuilt results.

Figure 6 shows the calculated spectral ratio curves of experimental result and rebuilt results. In the experimental result, the influence of blood was evenly distributed across the tissues, but in the rebuilt results, the influence of blood is mainly concentrated in the upper blood plexus, deep blood plexus and BCC layers. So the rebuilt results and the *in vivo* measurements have little difference at 540 nm and 580 nm. BCC layer with 2% blood added inside has a smaller influence on the final result than BCC layer with 5% blood, so 2% blood added in the BCC layer fits better at 540 nm and 580 nm. But to the whole curves, it can be seen that if 5% blood factor is added to the BCC layer, the reconstruct reflectance spectral ratios was more similar with the experimental result. If no blood factor added to the BCC layer, the reflectance spectral ratio have a big difference with *in vivo* measurement below 600 nm. Therefore, to build the BCC skin optical model, we need to take blood content into consideration during the simulation.

## 4. Conclusions

In this theoretical work, diffuse reflectance spectra of BCC skin were studied by the Monte Carlo method. We constructed an eight-layered optical model of BCC skin. By the Monte Carlo simulation, the light distribution was calculated in the model, and the diffuse reflectance process was modeled in order to study the effect of blood content of BCC layer on diffuse reflectance spectra. The ratios of the reflectance spectra in different skin models were discussed. When there is 5% blood factor added to the BCC layer, the reconstructed reflectance spectral ratios calculated by MC simulation were in a good agreement with the experimental results. Therefore, we should consider the blood content added to the BCC layer during this modeling work on BCC skin tissues. The reflectance spectral ratio methods and MC simulation may also be helpful for other cutaneous optical studies of the skin.

## Figures and Tables

**Table 1:** The optical parameters (*μ*_*a*_, *μ*_*s*_, *g*, *n*) at 442 nm of the seven-layer skin optical model.

Layer	*d* (*μ*m)	*n*	*μ*_*a*_ (cm^−1^)	*μ*_*s*_ (cm^−1^)	*g*
Air	—	1.0	—	—	—
Stratum corneum	10	1.45	207.4	580	0.7482
Epidermis	80	1.4	58.2	580	0.7482
Papillary dermis	100	1.4	6.72	681.5	0.7482
Upper blood plexus	80	1.39	97.26	654.35	0.7709
Reticular dermis	1500	1.4	6.72	681.5	0.7482
Deep blood plexus	70	1.34	821.58	518.15	0.9567
Dermis	160	1.4	6.72	681.5	0.7482
Subcutaneous fat	—	1.46	—	—	—

**Table 2:** The optical parameters (*μ*_*a*_, *μ*_*s*_, *g*, *n*) at 442 nm of the eight-layer BCC skin optical model.

Layer	*d* (*μ*m)	*n*	*μ*_*a*_ (cm^−1^)	*μ*_*s*_ (cm^−1^)	*g*
Air	—	1.0	—	—	—
Stratum corneum	10	1.45	207.4	580	0.7482
Epidermis	80	1.4	58.2	580	0.7482
Papillary dermis	100	1.4	6.72	681.5	0.7482
Upper blood plexus	80	1.39	97.26	654.35	0.7709
BCC layer	400	1.4	6.03	206.78	0.8
Reticular dermis	1100	1.4	6.72	681.5	0.7482
Deep blood plexus	70	1.34	821.58	518.15	0.9567
Dermis	160	1.4	6.72	681.5	0.7482
Subcutaneous fat	—	1.46	—	—	—

**Table 3:** The optical parameters (*μ*_*a*_, *μ*_*s*_, *g*, *n*) at 442 nm of the eight-layer BCC skin optical model. 2% blood factors added to the BCC layer.

Layer	*d* (*μ*m)	*n*	*μ*_*a*_ (cm^−1^)	*μ*_*s*_ (cm^−1^)	*g*
Air	—	1.0	—	—	—
Stratum corneum	10	1.45	207.4	580	0.7482
Epidermis	80	1.4	58.2	580	0.7482
Papillary dermis	100	1.4	6.72	681.5	0.7482
Upper blood plexus	80	1.39	97.26	654.35	0.7709
BCC layer	400	1.4	24.15	212.64	0.8
Reticular dermis	1100	1.4	6.72	681.5	0.7482
Deep blood plexus	70	1.34	821.58	518.15	0.9567
Dermis	160	1.4	6.72	681.5	0.7482
Subcutaneous fat	—	1.46	—	—	—

**Table 4:** The optical parameters (*μ*_*a*_, *μ*_*s*_, *g*, *n*) at 442 nm of the eight-layer BCC skin optical model. 5% blood factors added to the BCC layer.

Layer	*d* (*μ*m)	*n*	*μ*_*a*_ (cm^−1^)	*μ*_*s*_ (cm^−1^)	*g*
Air	—	1.0	—	—	—
Stratum corneum	10	1.45	207.4	580	0.7482
Epidermis	80	1.4	58.2	580	0.7482
Papillary dermis	100	1.4	6.72	681.5	0.7482
Upper blood plexus	80	1.39	97.26	654.35	0.7709
BCC layer	400	1.4	51.33	221.44	0.8
Reticular dermis	1100	1.4	6.72	681.5	0.7482
Deep blood plexus	70	1.34	821.58	518.15	0.9567
Dermis	160	1.4	6.72	681.5	0.7482
Subcutaneous fat	—	1.46	—	—	—

## References

[1] Chen R, Huang Z, Lui H (2007). Monte Carlo simulation of cutaneous reflectance and fluorescence measurements—the effect of melanin contents and localization. *Journal of Photochemistry and Photobiology B*.

[2] Zonios G, Dimou A (2006). Modeling diffuse reflectance from semi-infinite turbid media: application to the study of skin optical properties. *Optics Express*.

[3] Madan V, Lear JT, Szeimies R (2010). Non-melanoma skin cancer. *The Lancet*.

[4] Carr RA, Taibjee SM, Sanders DSA (2007). Basaloid skin tumours: basal cell carcinoma. *Current Diagnostic Pathology*.

[5] Telfer NR, Colver GB, Bowers PW (1999). Guidelines for the management of basal cell carcinoma. *British Journal of Dermatology*.

[6] Salomatina E, Jiang B, Novak J, Yaroslavsky AN (2006). Optical properties of normal and cancerous human skin in the visible and near-infrared spectral range. *Journal of Biomedical Optics*.

[7] Zeng H, MacAulay CE, Palcic B, McLean DI (1994). Monte Carlo modeling of tissue autofluorescence measurement and imaging. *The International Society for Optical Engineering*.

[8] Wang L, Jacques SL, Zheng L (1995). MCML—Monte Carlo modeling of light transport in multi-layered tissues. *Computer Methods and Programs in Biomedicine*.

[9] Zeng H, MacAulay C, McLean DI, Palcic B (1997). Reconstruction of in vivo skin autofluorescence spectrum from microscopic properties by Monte Carlo simulation. *Journal of Photochemistry and Photobiology B*.

[10] Wang S, Zhao J, Lui H, He Q, Zeng H (2011). Monte Carlo simulation of near infrared autofluorescence measurements of in vivo skin. *Journal of Photochemistry and Photobiology B*.

[11] Zeng H, MacAulay EC, McLean DI, Lui H, Palcic B Miniature spectrometer and multispectral imager as a potential diagnostic aid in dermatology.

[12] Zeng H, MacAulay C, McLean DI, Palcic B (1993). Novel microspectrophotometer and its biomedical applications. *Optical Engineering*.

[13] Graaf R, Dassel ACM, Koelink MH, de Mul FFM, Aarnoudse JG (1993). Optical properties of human dermis in vitro and in vivo. *Applied Optics*.

[14] Jacques SL, Rastegar S, Motamedi M Liver photocoagulation with diode laser (805 nm) versus Nd:YAG laser (1064 nm).

[15] Wang L, Jacques SL, Zheng L (1997). CONV—convolution for responses to a finite diameter photon beam incident on multi-layered tissues. *Computer Methods and Programs in Biomedicine*.

